# Human and Animal Blood

**Published:** 1892-06

**Authors:** 


### Human and Animal Blood.

Dr. Pesety Cevera claims that human may be distinguished from animal blood by the following method: If the blood be mixed with a little bile, small crystals are formed which are of different shapes in different species of animals. In man, it is claimed, they are right-angled prisms; in the horse cubes; in pigs right-angled prisms, very similar to those seen in rhomboids; in sheep rhomboidal plates; in dogs the same as seen in human blood; in chickens more or less regular cubes.

